# The Diagnostic Challenge of Crohn's Disease With Initial Duodenal Involvement: Two Cases and Literature Review

**DOI:** 10.1002/iid3.70491

**Published:** 2026-07-27

**Authors:** Chaochao Chen, Yongrong Li, Yiyao Chen, Zhoutao He, Cheng Lan

**Affiliations:** ^1^ Department of Gastroenterology, Hainan General Hospital Hainan Affiliated Hospital of Hainan Medical University Haikou China

**Keywords:** Crohn's disease, delay, diagnosis, duodenal

## Abstract

**Background:**

Upper gastrointestinal Crohn's disease (UGI‐CD) is rare, with an adult prevalence of 0.5%–4.0%. Its nonspecific symptomatology and the absence of clear diagnostic guidelines pose significant diagnostic challenges.

**Case Presentation:**

We present two patients with UGI‐CD treated at Hainan General Hospital. In the first case, a 47‐month diagnostic delay led to disease progression and surgical intervention. In the second case, prompt diagnosis of a duodenal ulcer enabled medical management, avoiding surgery. Both patients received infliximab and achieved remission.

**Conclusion:**

These cases highlight diagnostic challenges in UGI‐CD and offer three key lessons: (i) UGI‐CD should be suspected in refractory, *Helicobacter pylori*‐negative duodenal ulcers; (ii) an endoscopic‐radiologic mismatch may occur; and (iii) we propose a diagnostic algorithm to assist early recognition, though it has not been prospectively validated. Given the descriptive nature of this two‐case series, these findings are hypothesis‐generating and require further validation.

## 1 Introduction

Inflammatory bowel disease (IBD), primarily comprising ulcerative colitis and Crohn's disease (CD), is a chronic relapsing condition [1]. CD was first described in 1932 and can affect any part of the gastrointestinal tract [2]. The terminal ileum and colon are the most common sites of involvement [3]. Upper gastrointestinal (UGI) tract involvement, particularly in the duodenum, was once considered rare 0.5 [4]. However, recent evidence suggests a higher prevalence, with incidence rates up to 11.5% [5].

Despite this increased recognition, UGI‐CD remains a formidable diagnostic challenge. This is due to its non‐specific symptoms and endoscopic mimics. Consequently, this case series and literature review aim to elucidate the common diagnostic pitfalls of UGI‐CD. We also propose strategies to mitigate diagnostic delay.

This diagnostic conundrum stems from several factors. First, many patients with CD and duodenal involvement are asymptomatic or present with non‐specific symptoms. As a result, isolated cases or those with duodenal involvement as the initial manifestation are frequently misdiagnosed as common duodenal ulcers [6, 7]. Second, there are no universally accepted diagnostic criteria. Although features such as contiguous disease in the distal small bowel and characteristic endoscopic findings (e.g., skip lesions, cobblestoning) are considered suggestive, their inconsistency and low sensitivity often lead to diagnostic uncertainty and significant delay [8, 9]. These formidable obstacles underscore the critical need to improve clinical recognition. This is the primary aim of presenting these cases and reviewing the pertinent literature.

Several case reports have described duodenal involvement in CD. However, few have quantified the duration of diagnostic delay. Few have addressed the specific mismatch between endoscopic stenosis and cross‐sectional imaging. Furthermore, a structured approach to diagnosing UGI‐CD in patients presenting with refractory duodenal ulcers remains lacking. This paper reports two contrasting cases of duodenal‐predominant CD. We emphasize an extreme 47‐month delay, an endoscopic‐radiologic mismatch, and a proposed diagnostic algorithm. This algorithm aims to reduce delayed recognition and improve clinical outcomes.

### 1.1 Literature Search Strategy

A literature search was performed using PubMed, Web of Science, and Google Scholar databases, using the following search terms: “upper gastrointestinal Crohn's disease,” “duodenal Crohn's disease,” “diagnostic delay,” and “biologic therapy.” References of relevant articles were manually screened for additional studies.

## 2 Cases Presentation

### 2.1 Case Selection

These two cases were selected from our clinical practice because they represent opposite ends of the diagnostic spectrum in UGI‐CD. Case 1 illustrates the extreme consequence of diagnostic delay (47 months), leading to irreversible stricture and surgical resection. Case 2 demonstrates the favorable outcome achievable with early recognition and timely biologic therapy. Together, they provide a contrastive teaching model that highlights the critical importance of early diagnosis. While two cases cannot establish generalizable estimates, they serve to illustrate specific diagnostic pitfalls—namely, the failure to suspect CD in refractory duodenal ulcers (Case 1) and the value of a low threshold for small‐bowel imaging and biopsy (Case 2).

### 2.2 Case 1

**Figure 1:**
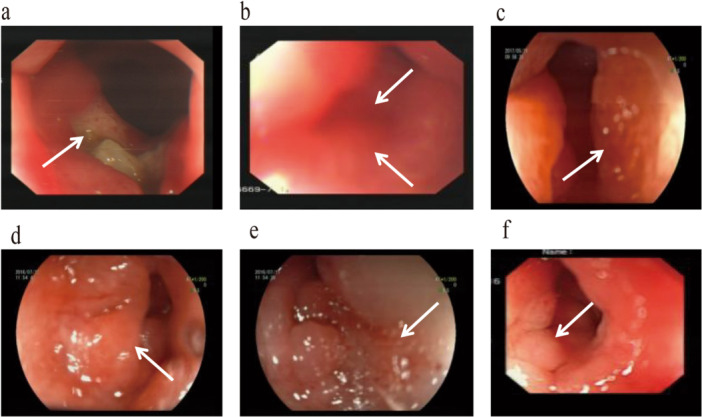
Endoscopic findings in Case 1: Giant duodenal ulcer with stenosis and cobblestone appearance. Gastroscopic images show multifocal duodenal ulcers, severe stenosis, and nodular/cobblestone mucosa, with distal extension beyond the bulb and *Helicobacter pylori* negativity. Arrows indicate key lesions: (a) ulcer, (b) stenosis, (c–f) cobblestone mucosa.

A previously healthy 55‐year‐old man presented to Hainan General Hospital in February 2016, complaining of epigastric pain. Blood tests showed a hemoglobin level of 10.5 g/100 mL, C‐reactive protein at 4.15 mg/L, and an erythrocyte sedimentation rate of 22 mm/h. Both the *Helicobacter pylori* stool antigen test and T‐Sport were negative. A chest CT scan revealed no significant abnormalities. He denied vomiting, melena, or long‐term NSAID use but had a history of small bowel resection and smoking. An initial gastroscopy revealed a giant *H. pylori*‐negative ulcer with stenosis in the duodenal bulb (Figure 1). Despite a diagnosis of duodenal ulcer and prolonged trials of rabeprazole and esomeprazole, his symptoms (epigastric pain and early satiety) persisted and recurred after brief periods of relief.

Over the subsequent 3 years, the patient underwent six additional gastroscopies. All examinations confirmed persistent duodenal ulceration and stenosis. However, no biopsy was performed for two main reasons: first, the severe luminal stenosis prevented the endoscope from advancing, and a biopsy would have posed a significant risk of bleeding or perforation; second, the patient's history was consistent with recurrent ulcers, and he was therefore managed with standard ulcer therapy without further investigation for CD at that time.

**Figure 2:**
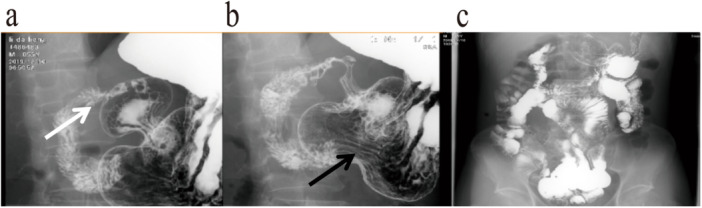
Alimentary tract contrast imaging in Case 1: Dual involvement of duodenum and ileum. Contrast radiography reveals two distinct abnormalities: (a) duodenal ulceration and stricture in the duodenal bulb (white arrow), corresponding to the endoscopic findings shown in Figure 1; (b) ileal stenosis in the distal ileum (black arrow); and (c) multiple intestinal lesions in the duodenum and distal ileum.

**Figure 3:**
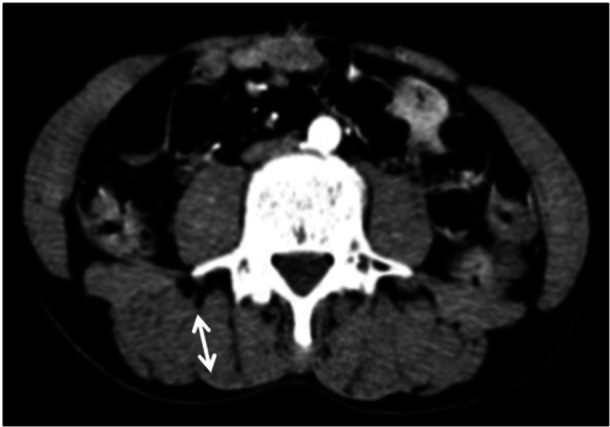
CT imaging in Case 1: Subtle abnormality despite severe endoscopic findings. CT scan of the abdomen shows a soft tissue nodule in the left lower abdomen (white arrow). Differential considerations included inflammatory cellulitis, granuloma, or tumor.

In December 2019, he was admitted with worsening symptoms, including forceful vomiting, weight loss, and succussion splash. Alimentary tract contrast imaging revealed duodenal ulceration and stricture alongside ileal stenosis (Figure 2). A CT scan showed a soft‐tissue nodule in the left lower abdomen, with differential considerations including inflammatory cellulitis, granuloma, or tumor, accompanied by signs of incomplete intestinal obstruction (Figure 3). Surgical intervention was required: a distal subtotal gastrectomy, gastrojejunostomy (BII anastomosis), release of intestinal adhesions, and partial small bowel resection. Intraoperative findings and histology of resected specimens showed cobblestoning and transmural inflammation with lymphoid aggregates, confirming CD. Specimens were reviewed by two independent pathologists and reported according to standardized histopathological criteria. The patient was started on infliximab postoperatively, made an uneventful recovery, and remains in good health (Table 1).

**Table 1:** Diagnosis timeline for Case 1.

Date	Symptoms	Investigations	Key conclusions	Interventions	Outcome
February 2016	Epigastric hunger pain, worse at night, radiating to the back	Gastroscopy; *H. pylori* test	Giant ulcer covering entire duodenal bulb (Figure 1); *H. pylori* negative	Rabeprazole	No improvement
March 2016	Persistent symptoms	Repeat gastroscopy	Persistent stenosis at bulboduodenal junction (Figure 1)	Esomeprazole (3 months)	Brief relief; recurrence after discontinuation
July 2016	Early satiety; pain recurrence	Third gastroscopy	Persistent duodenal stenosis (Figure 1)	Esomeprazole; semi‐liquid diet	Symptoms not fully resolved
May 2017–April 2019	Persistent abdominal pain and early satiety	Four additional gastroscopies	Persistent duodenal ulcers and strictures; no biopsies taken	Continuous PPI therapy	Symptoms not fully resolved
December 2019	Worsening epigastric pain, foul‐smelling eructations, projectile vomiting, weight loss; succussion splash	Gastrointestinal contrast imaging; CT	Duodenal ulceration and stricture; distal ileal stenosis (Figure 2); soft‐tissue nodule in left lower abdomen; signs of incomplete intestinal obstruction (Figure 3)	Surgical intervention; post‐operative infliximab	Uneventful recovery; symptom resolution; remains well on follow‐up

*Note:* This table summarizes the 47‐month diagnostic delay in Case 1. Key teaching points include: (i) despite persistent symptoms and endoscopic findings suggestive of CD (giant ulcer, stenosis), no biopsies were obtained during repeated endoscopies; (ii) the absence of typical CD features on initial CT falsely reassured clinicians; and (iii) diagnostic delay led to irreversible stricture and surgical intervention.

Abbreviations: CD, Crohn's disease; CT, computed tomography; *H. pylori*, *Helicobacter pylori*; PPI, proton pump inhibitor; UGI‐CD, upper gastrointestinal Crohn's disease.

### 2.3 Case 2

**Figure 4:**
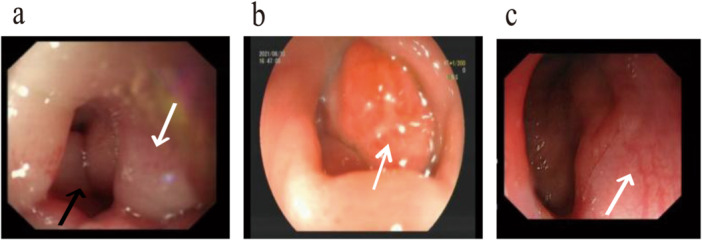
Endoscopic findings in Case 2: Duodenal ulcer with stricture. Gastroscopy of the duodenum reveals a duodenal ulcer (white arrow) accompanied by a luminal stricture (black arrow). (a) endoscopic view of the duodenal bulb: black arrow indicates a segmental luminal stricture; white arrow points to the adjacent ulcer; (b) magnified endoscopic image of the ulcer (white arrow); and (c) distal duodenal endoscopic view showing congested and edematous mucosa surrounding the stricture and ulcer (white arrow).

**Figure 5:**
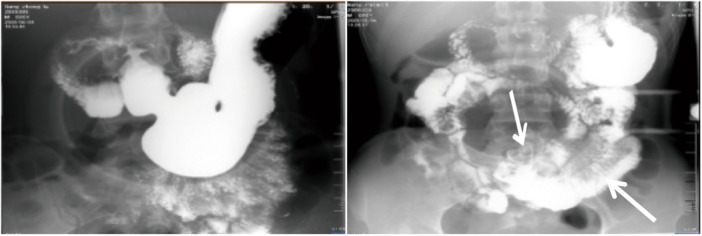
Small‐bowel imaging in Case 2: Multiple segmental strictures. Barium radiograph/enterography demonstrates multiple segmental strictures in the small intestine (white arrows).

**Figure 6:**
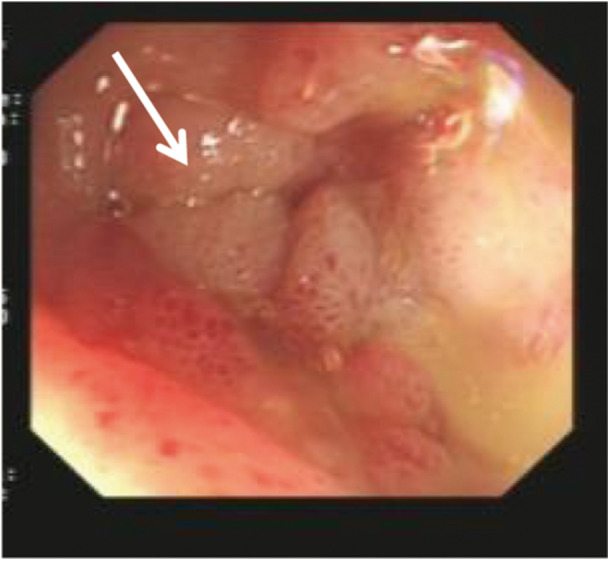
Colonoscopy in Case 2: Nodular protuberances at the terminal ileum. Colonoscopy reveals nodular protuberances at the terminal ileum (white arrows).

A previously healthy 26‐year‐old man was admitted to Hainan General Hospital in August 2020 for epigastric pain of 20 days' duration and vomiting for 3 days. His medical history was unremarkable except for anal fistula surgery in 2019. Physical examination revealed epigastric tenderness as the only abnormal finding. Blood tests showed a hemoglobin level of 12.6 g/100 mL, C‐reactive protein at 10 mg/L, and an erythrocyte sedimentation rate of 38 mm/h. Both T‐Sport and *H. pylori* tests were negative. A chest CT scan did not reveal any abnormalities. Gastroscopy identified a duodenal ulcer accompanied by a stricture (Figure 4). A barium radiograph indicated that the stomach was enlarged and contained excess fluid, with deformation of the duodenal bulb and a probable ulcer crater causing some obstruction. Multiple segmental strictures were also detected in the small intestine (Figure 5). These findings strongly suggested a diagnosis of UGI‐CD. Consequently, a colonoscopy was performed, revealing nodular protuberances at the terminal ileum (Figure 6). A biopsy of the terminal ileum confirmed the presence of non‐caseating granulomas. Follow‐up evaluation included symptom assessment, laboratory monitoring, and repeat imaging at 3–6 month intervals.

The patient was clinically diagnosed with UGI‐CD and subsequently treated with infliximab. This treatment led to recovery, allowing him to avoid surgery. Following the initiation of infliximab therapy, the patient's course was complicated by a disease flare in August 2021, requiring hospitalization for obstructive symptoms. This episode was successfully managed with conservative measures (fasting, decompression) and optimization of his immunosuppressive regimen. Subsequently, he was maintained on a combination therapy of infliximab and azathioprine. On this regimen, he achieved and has maintained sustained clinical remission for over 24 months of follow‐up, with no further hospitalizations, resolution of nausea and vomiting, and successful weight regain. Regular infusions have been well‐tolerated (Table 2).

**Table 2:** Diagnosis timeline for Case 2.

Date	Symptoms	Investigations	Key conclusions	Interventions	Outcome
August 2020	Upper abdominal pain (20 days); vomiting (3 days)	*H. pylori* test; chest CT; gastroscopy; upper gastrointestinal barium contrast examination; colonoscopy with terminal ileum biopsy	*H. pylori* negative; chest CT unremarkable; duodenal ulcer with stricture (Figure 4); barium radiography: gastric dilatation with fluid stasis, duodenal bulb deformity, multiple segmental small‐bowel strictures (Figure 5); colonoscopy: nodular protrusions at terminal ileum; histopathology: granulomas confirmed	Infliximab	Clinical remission achieved; surgery avoided
August 2021	Abdominal distension, nausea, vomiting	Abdominal X‐ray; endoscopy	Upper gastrointestinal stenosis; anal fistula; gastric ulcer; pyloric obstruction	Azathioprine; infliximab; lansoprazole	Symptom resolution

*Note:* This table summarizes the prompt diagnosis and successful outcomes in Case 2. Key teaching points include: (i) early cross‐sectional imaging revealed small‐bowel strictures despite moderate endoscopic findings; (ii) ileal granulomas on biopsy confirmed the diagnosis; and (iii) timely infliximab therapy induced remission and avoided surgery.

Abbreviations: CD, Crohn's disease; CT, computed tomography; *H. pylori*, *Helicobacter pylori*; UGI‐CD, upper gastrointestinal Crohn's disease.

## 3 Literature Review and Discussion

### 3.1 Epidemiology

Since Gottlieb and Alpert first described UGI‐CD in 1937 [10], it has been increasingly recognized. One study reported a prevalence of 6.5% for UGI‐CD at the time of diagnosis [11]. However, a systematic review found a higher overall prevalence of 16.6% [12]. Improved imaging modalities likely account for the increased prevalence, as they have significantly enhanced the detection of small bowel lesions.

However, studies have shown varying rates of UGI involvement in CD. Chin et al. found that 13% of CD patients exhibited UGI‐CD [13], while another study using combined UGI endoscopy and colonoscopy reported UGI involvement in 41% of newly diagnosed adult CD patients, with endoscopic lesions present in 55% of cases [14]. These discrepancies likely arise from differences in diagnostic criteria, study methodologies, patient selection, and the routine use of upper endoscopy. Despite these variations, current evidence consistently indicates a rising prevalence of UGI‐CD.

When contextualizing our cases within this epidemiological landscape, it becomes evident that they contribute to the growing body of literature on this rare condition. The historical perception of UGI‐CD as an exceedingly rare entity is increasingly being challenged by modern data, as reflected in our review. The patients presented in this report exemplify the diagnostic challenges that persist even as recognition of the disease improves.

### 3.2 Clinical Presentation

The most frequent symptom of UGI‐CD is postprandial dyspepsia. Other common manifestations include nausea, anorexia, diarrhea, and weight loss [15, 16]. In cases complicated by gastric outlet obstruction, patients may experience early satiety, postprandial vomiting, significant weight loss, or, rarely, hematemesis [17]. Less commonly, involvement of the papilla of Vater can lead to biliary colic, while duodenal inflammation may result in pancreatitis or hemorrhage due to vascular erosion [18]. Importantly, these symptoms are often misattributed to peptic ulcer disease or adverse effects of medications [6]. This non‐specific symptomatic profile contributes to considerable diagnostic challenges.

Both patients in our report presented with refractory upper abdominal pain resembling that of peptic ulcer disease. Their poor response to conventional therapies and the subsequent development of obstructive symptoms underscore the diagnostic difficulties described in the literature and highlight the necessity of maintaining a high index of suspicion for CD in such clinical scenarios.

### 3.3 Endoscopic and Radiological Features

#### 3.3.1 Endoscopic Findings

Endoscopic features of UGI‐CD include mucosal nodularity, cobblestoning, fold notching, stenosis, longitudinal or linear ulcers, and a characteristic “cobblestone appearance” (deep, intersecting ulcers separating islands of edematous mucosa, resembling a cobblestone street) [8, 19]. While some findings (e.g., edema, aphthous ulcers) are nonspecific [20], features such as serpiginous ulcers, cobblestoning, and duodenal stenosis are more specific to CD.

As illustrated in Figure 1 (Case 1), the initial gastroscopy revealed a giant, *H. pylori*‐negative duodenal bulb ulcer with significant luminal stenosis. Several endoscopic features in this case are worth highlighting as teaching points. First, the ulcer demonstrated a longitudinal configuration rather than the round or oval shape typical of benign peptic ulcer disease. Second, the presence of luminal stenosis in a young patient with refractory symptoms is another red flag. Third, the cobblestone appearance of the surrounding mucosa is considered relatively specific for CD [19]. Despite these suggestive findings, the diagnosis was delayed for 47 months in Case 1, largely because deep biopsies were not obtained during repeated endoscopic procedures. Therefore, when endoscopic features suggestive of CD are observed (e.g., longitudinal ulcers, stenosis, cobblestoning), deep biopsies must be obtained during every endoscopic procedure, regardless of whether the primary intent is diagnostic or therapeutic.

As illustrated in Figure 4 (Case 2), endoscopic findings were initially interpreted as a benign duodenal ulcer attributed to NSAID use. However, the presence of concomitant small‐bowel strictures on cross‐sectional imaging (discussed below) prompted reconsideration of the diagnosis. This comparison between the two cases highlights that endoscopic severity does not necessarily correlate with the extent of small‐bowel involvement, and that mild endoscopic findings should not lower suspicion for CD when other features (e.g., refractory symptoms, young age, weight loss) are present.

#### 3.3.2 Cross‐Sectional Imaging Features

UGI‐CD is often evaluated following cross‐sectional imaging, including CT, CT enterography (CTE), and MR enterography (MRE). CT/CTE effectively identifies wall thickening, stenosis, fistulas, abscesses, and extraluminal complications but involves ionizing radiation [21]. MRE is radiation‐free and excels in depicting inflammation and penetrating complications [22, 23], though its sensitivity for detecting stenosis requires further study [24]. Both modalities help evaluate disease extent and complications, supporting diagnosis and management.

The imaging findings in our two cases illustrate a critical diagnostic challenge: the endoscopic‐radiologic mismatch. In Case 1, endoscopy revealed severe duodenal stenosis with a cobblestone appearance, yet initial CT showed only a soft tissue nodule without typical features of CD (e.g., mural thickening, fat stranding, or fistulae) (Figure 3). This mismatch contributed to the 47‐month diagnostic delay, as clinicians were falsely reassured by the negative imaging findings. Notably, alimentary tract contrast imaging (Figure 2) was more revealing in Case 1, demonstrating both duodenal and ileal involvement. This suggests that when CT is equivocal, alternative imaging modalities such as contrast radiography or MRE may provide additional diagnostic information. In contrast, in Case 2, cross‐sectional enterography demonstrated small‐bowel strictures even though endoscopic changes were initially considered moderate (Figure 5). This case illustrates that imaging may reveal small‐bowel involvement even when duodenal findings are subtle.

The absence of typical CD features on cross‐sectional imaging does not exclude UGI‐CD. When endoscopic findings are suspicious (e.g., longitudinal ulcers, stenosis, cobblestoning) but imaging is negative, clinicians should maintain a high index of suspicion and proceed with deep biopsies or advanced small‐bowel evaluation (capsule endoscopy or balloon‐assisted enteroscopy). Conversely, when imaging reveals small‐bowel strictures in a patient with upper GI symptoms, CD should be strongly suspected even if endoscopic findings appear mild. As demonstrated in Case 1 (Figure 2), contrast radiography can be a useful adjunct when CT findings are equivocal.

The experience from our two cases underscores the ECCO consensus principle that multimodal diagnostic approaches—combining endoscopy with deep biopsies and cross‐sectional imaging—are essential for accurate diagnosis and characterization of UGI‐CD, particularly in cases where initial findings are ambiguous or non‐specific. Neither endoscopy nor imaging alone is sufficient; the diagnosis requires integration of all available data.

### 3.4 Endoscopic‐Radiologic Mismatch as a Diagnostic Pitfall

A striking observation in our case series is the mismatch between endoscopic findings and initial radiologic imaging. In Case 1, endoscopy revealed a severe duodenal ulcer with high‐grade stenosis, yet early computed tomography did not show typical features of CD, such as mural thickening, fat stranding, or fistulae. This mismatch contributed to a 47‐month diagnostic delay. In contrast, in Case 2, cross‐sectional enterography demonstrated small‐bowel strictures even though endoscopic changes were initially considered moderate. We propose that in patients with a refractory, *H. pylori*‐negative duodenal ulcer with stenosis, the absence of classic imaging findings does not exclude UGI‐CD. When endoscopic and imaging findings diverge, clinicians should lower the threshold for endoscopic re‐biopsy, small‐bowel capsule endoscopy, or magnetic resonance enterography.

### 3.5 Histology

Endoscopy with histology is considered the gold standard for diagnosing UGI‐CD. One study demonstrated that among patients with UGI‐CD, 84% exhibited gastric inflammation on histopathology, followed by duodenal inflammation in 28.2% and gastric granulomas in 23.2% of cases [25]. The sensitivity of endoscopic biopsy for detecting granulomas in the duodenum is notoriously low, with reported rates ranging from 3.4% to 11.1% [26, 27]. Histology may reveal granulomas or transmural lymphoid aggregates, both of which are pathognomonic for CD; however, it is important to note that their absence does not exclude the diagnosis [28]. In a study of 225 CD patients who underwent UGI endoscopy, duodenal lesions were detected in 34% of cases [26], but granulomas were identified in only 3.4% [27]. Therefore, to exclude alternative diagnoses, multiple biopsies should be taken from different areas of the duodenum during endoscopy. The results should then be integrated with the patient's clinical context to support a comprehensive diagnosis.

The histological findings in our cases vividly illustrate the challenges and necessities described in the literature. In both patients, biopsies were obtained from the duodenum. The biopsy from Patient 1 revealed significant transmural inflammation penetrating the intestinal wall. Although this finding alone is not sufficient to establish a definitive histological diagnosis, it strongly suggests CD within the appropriate clinical and endoscopic context and effectively rules out other differential diagnoses. In Patient 2, granulomas were identified, which are a characteristic pathological feature of CD. The comparison between these two cases highlights the importance of the integration of clinical, endoscopic, and histological aspects in the diagnosis of UGI‐CD. Importantly, when biopsies are negative, a systematic approach to further evaluation is required, as outlined below.

Step 1: Re‐evaluate clinical suspicion. Persistent refractory symptoms (e.g., no response to 8–12 weeks of high‐dose PPI), alarm features (weight loss, anemia, young age), or the presence of extra‐intestinal manifestations should maintain suspicion for CD even with negative biopsies. Step 2: Repeat endoscopy with deeper and more extensive biopsies. If initial biopsies were superficial, limited in number (< 6), or taken only from ulcer centers rather than margins, repeat endoscopy with ≥ 6 deep biopsies from multiple sites (duodenal bulb, second part, and normal‐appearing mucosa) is recommended. Step 3: Obtain cross‐sectional imaging (CTE or MRE). Imaging may reveal small‐bowel involvement (wall thickening, strictures, fistulae) even when duodenal biopsies are negative. In Case 2, ileal strictures on MRE prompted the diagnosis despite non‐diagnostic duodenal histology initially. Step 4: Consider small‐bowel capsule endoscopy or balloon‐assisted enteroscopy. If upper endoscopy and imaging are unrevealing but suspicion remains high, capsule endoscopy can identify proximal small‐bowel lesions not reached by standard endoscopy. Step 5: Clinical and endoscopic follow‐up. In equivocal cases, repeat endoscopy with biopsies after 3–6 months may reveal diagnostic features that were initially absent.

Negative duodenal biopsies should never be used as the sole criterion to exclude UGI‐CD. Instead, they should prompt a systematic re‐evaluation combining repeat endoscopy, cross‐sectional imaging, and, when necessary, small‐bowel enteroscopy. The diagnosis of UGI‐CD ultimately relies on the integration of clinical, endoscopic, radiographic, and histological findings—not on any single negative test.

### 3.6 Differential Diagnoses

The clinical manifestations of duodenal tuberculosis can include weight loss, fever, hematemesis, pyloric obstruction, and involvement of other parts of the gastrointestinal tract [29]. However, UGI‐CD can be distinguished by the absence of systemic symptoms (e.g., fever or night sweats), a negative interferon‐gamma release assay, and no caseous granuloma on histopathology.

Gastrointestinal lymphoma, a relatively rare tumor accounting for 1% to 8% of gastrointestinal malignancies [30], was also considered. Although it can present with similar ulcerative and infiltrative lesions, features such as unexplained high fever, elevated LDH and β2‐microglobulin levels, and a biopsy revealing a diffuse infiltrate of monotonous lymphocytes are atypical for UGI‐CD [31]. Therefore, this possibility was excluded.

Refractory peptic ulcer disease was an initial consideration, but the failure to respond to high‐dose proton pump inhibitor therapy, the atypical location and morphology of the ulcers (e.g., longitudinal), and the presence of chronic inflammation and architectural distortion on histology argued against this diagnosis.

Celiac disease is an immune‐mediated disease caused by gluten, and its diagnosis relies on positive antibodies (adult anti‐transglutaminase and child anti‐gliadin) and duodenal biopsy (villous atrophy and increased number of intraepithelial lymphocytes) [32]. In UGI‐CD, these serological and histological markers for celiac disease are negative; therefore, concomitant celiac disease (e.g., with refractory duodenitis) was also excluded.

### 3.7 Diagnostic Delay

The diagnosis of UGI‐CD is frequently delayed due to several interrelated factors. First, the condition often presents with a prolonged prodromal phase dominated by non‐specific abdominal symptoms, leading to frequent misdiagnosis such as irritable bowel syndrome [33]. Second, a significant proportion of patients remain asymptomatic in early stages [34], resulting in a typical diagnostic delay ranging from 5 to 34 months after symptom onset [35]; this subclinical presentation hinders detection through routine endoscopy. Third, isolated or primary UGI‐CD is commonly mistaken for other pathologies such as duodenal ulcer [36]. These diagnostic challenges are clearly illustrated in Case 1, where the diagnosis was delayed by 47 months.

Delayed diagnosis in CD is associated with a significantly elevated risk of complications and greater treatment difficulty. Studies indicate that when diagnosis is delayed beyond 26 months, the complication rate reaches 63%, compared to only 25% among those diagnosed within 4 months of symptom onset [37, 38]. This trend is clearly illustrated in our Case 1, where stenosis had already developed by the time of diagnosis.

The diagnostic delay in Case 1, exacerbated by the omission of biopsies during repeated endoscopies, illustrates a critical clinical reasoning failure. From a clinical standpoint, several factors likely contributed to this error. First, the endoscopic finding of duodenal stenosis may have created a technical barrier, leading the endoscopist to prioritize passage of the scope over tissue acquisition. However, even in the setting of severe stenosis, biopsies should be attempted from the ulcer margin or adjacent mucosa. Second, the absence of typical CD features on initial computed tomography may have falsely reassured clinicians, reinforcing a diagnostic anchor of peptic ulcer disease. This represents a classic cognitive bias—premature closure. Third, repeated endoscopies were performed for therapeutic purposes (e.g., dilation) rather than diagnostic re‐evaluation.

We therefore critically note that every endoscopic procedure in a patient with a refractory, *H. pylori*‐negative duodenal ulcer should include deep biopsies, regardless of whether the primary intent is therapeutic. The failure to do so in Case 1 directly contributed to a 47‐month delay and subsequent surgery.

### 3.8 Analytical Approach to the Underlying Causes of Diagnostic Delay

While the above sections describe the occurrence and consequences of diagnostic delay in UGI‐CD, a deeper analytical approach is necessary to understand why such delays persist despite increasing disease recognition. Based on our cases and a critical review of the literature, we identify five key factors that systematically contribute to diagnostic delay in UGI‐CD:

1. Lack of standardized diagnostic guidelines. Unlike colonic CD, for which clear diagnostic algorithms exist, UGI‐CD lacks specific, evidence‐based guidelines [39, 40]. Current ECCO guidelines mention upper GI involvement but do not provide a step‐by‐step diagnostic approach for patients presenting with isolated duodenal ulcers.
2. Biopsy reluctance in the setting of duodenal stenosis. As illustrated in Case 1, the presence of duodenal stenosis creates both a technical and a cognitive barrier. Technically, passing an endoscope through a tight stenosis may be challenging. Cognitively, the visible stenosis may be mistakenly interpreted as a consequence of chronic peptic ulcer disease. We propose that stenosis should increase, not decrease, the suspicion for CD.
3. Diagnostic anchoring on resistant peptic ulcer disease. The initial diagnosis of peptic ulcer disease, once established, tends to persist even when treatment fails. This represents anchoring bias. In Case 1, despite 47 months of refractory symptoms, the diagnostic anchor of peptic ulcer was never seriously challenged.
4. Nonspecific and overlapping symptoms. The clinical presentation of UGI‐CD is virtually indistinguishable from that of peptic ulcer disease, gastroparesis, or functional dyspepsia. This symptomatic overlap means that CD is simply not considered in the initial differential diagnosis for most patients.
5. Low histological yield of granulomas in duodenal biopsies. Even when biopsies are obtained, the diagnostic yield for granulomas is notoriously low, ranging from 3.4% to 11.1% in published series [26, 27]. This means that a negative biopsy does not exclude CD.

These five factors—guideline gaps, biopsy reluctance, anchoring bias, symptom overlap, and low granuloma yield—operate synergistically to prolong diagnostic delay in UGI‐CD.

### 3.9 Clinical Implications of Diagnostic Delay

The natural history of CD commonly involves progression from inflammatory changes to stricturing or penetrating complications [41]. In particular, stricture formation represents one of the most frequent and serious complications in UGI‐CD. Therefore, prompt identification and timely intervention are critical to prevent irreversible bowel damage and reduce the need for surgical resection.

Adhering to the “early intervention” principle highlighted at the ECCO scientific symposium is essential [42]. Early immunosuppressive therapy, including biologics, has been shown to improve treatment response and reduce adverse outcomes [43, 44]. This approach is exemplified in our Case 2, in which prompt diagnosis and initiation of biologic therapy led to a significant clinical response and avoided surgical intervention.

### 3.10 Diagnostic Pitfalls Illustrated by the Two Cases

Case 1 demonstrates the pitfall of clinical inertia: despite a refractory, *H. pylori*‐negative duodenal ulcer with stenosis, CD was not considered for 47 months. Repeated endoscopic procedures were performed without obtaining deep biopsies, and the absence of typical IBD findings on initial imaging falsely reassured clinicians. From a clinical reasoning perspective, this case exemplifies two specific cognitive errors. First, anchoring bias—clinicians persisted with the initial diagnosis of peptic ulcer disease despite repeated treatment failure. Second, availability bias—the negative imaging finding was readily available and thus overweighted, while the refractory symptoms were underweighted. This case underscores that UGI‐CD can mimic benign peptic ulcer disease and that negative imaging does not exclude the diagnosis.

Case 2, in contrast, illustrates the pitfall of under‐recognition of upper GI involvement: the duodenal ulcer was initially attributed to NSAID use, but the presence of concomitant small‐bowel strictures on cross‐sectional imaging prompted timely consideration of CD. The key lesson is that duodenal ulcers should prompt evaluation of the entire small bowel, especially when they are recurrent or refractory to standard therapy.

### 3.11 Relationship to Prior Literature

Previous case series have reported duodenal involvement in 0.5%–4% of CD patients, often with diagnostic delays ranging from several months to years. However, few have quantified a delay as prolonged as 47 months, and none have explicitly described the endoscopic‐radiologic mismatch as a specific contributor to delayed diagnosis. Our Case 1 extends prior observations by demonstrating that even in the era of modern cross‐sectional imaging, a refractory duodenal ulcer without typical CD imaging features can remain undiagnosed for nearly 4 years.

Conversely, Case 2 aligns with recent reports advocating for early biologic therapy in UGI‐CD, but adds the specific recommendation that small‐bowel imaging should be performed early, even when endoscopic changes appear limited. Our Case 2 provides real‐world evidence that prompt recognition and timely infliximab initiation can alter disease trajectory and avoid surgery.

Thus, these two cases complement the existing literature in three ways: (i) they provide a contrastive illustration of delayed versus prompt diagnosis within a single report; (ii) they highlight an endoscopic‐radiologic mismatch phenomenon that has been underemphasized; and (iii) they offer a practical diagnostic algorithm grounded in both literature evidence and real‐world case experience.

### 3.12 Integration of Imaging Findings: Teaching Points From Figures 1, 2, 3, 4, 5, 6

The multimodal imaging findings presented in Figures 1, 2, 3, 4, 5, 6 reinforce several key teaching points that merit explicit discussion.

First, endoscopic morphology dictates diagnostic action. As shown in Figure 1 (Case 1), the longitudinal configuration of the duodenal ulcer and the cobblestone appearance of the surrounding mucosa are features that should raise immediate suspicion for CD. Therefore, when such endoscopic features are observed, deep biopsies must be obtained during every endoscopic procedure, regardless of whether the primary intent is diagnostic or therapeutic.

Second, upper GI involvement in CD is often not isolated. Figure 2 demonstrates concomitant duodenal and ileal involvement—discontinuous lesions separated by normal bowel—which is highly characteristic of CD. This finding underscores that patients with suspected UGI‐CD should undergo evaluation of the entire small bowel.

Third, the endoscopic‐radiologic mismatch is a critical diagnostic pitfall. As illustrated by the two cases, reliance on a single modality can be misleading. In Case 1, severe endoscopic findings (Figure 1) were accompanied by only a subtle CT abnormality (Figure 3). Conversely, in Case 2, mild endoscopic findings (Figure 4) were associated with significant small‐bowel strictures on enterography (Figure 5). Thus, the absence of typical CD features on cross‐sectional imaging does not exclude UGI‐CD, nor should mild endoscopic findings lower clinical suspicion when other features (e.g., refractory symptoms, young age) are present.

Fourth, alternative imaging modalities add value when the initial CT is equivocal. As demonstrated in Case 1 (Figure 2), alimentary tract contrast radiography revealed both duodenal and ileal involvement that was not apparent on initial CT, helping to resolve the diagnostic uncertainty.

**Figure 7:**
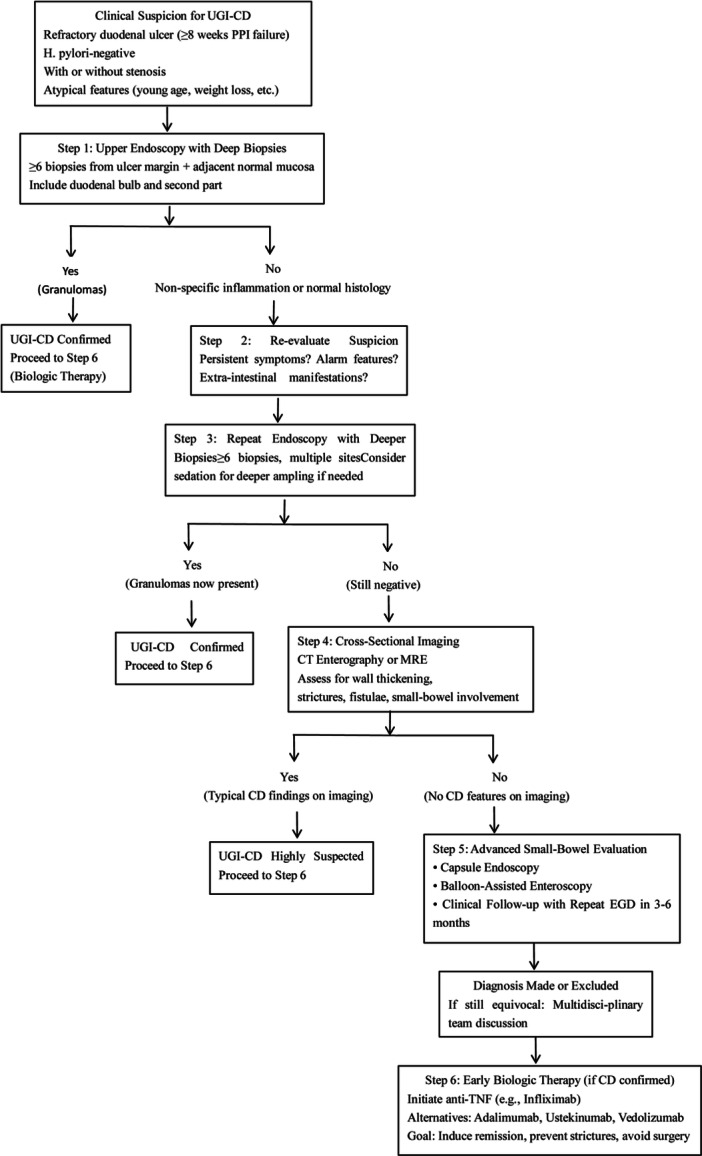
Diagnostic algorithm for suspected UGI‐CD in patients presenting with refractory duodenal ulcers. This algorithm represents a proposed diagnostic framework based on the authors' experience and literature review. It has not been prospectively validated and should be applied with clinical judgment. CD, Crohn's disease; CTE, computed tomography enterography; EGD, esophagogastroduodenoscopy; MRE, magnetic resonance enterography; PPI, proton pump inhibitor; UGI‐CD, upper gastrointestinal Crohn's disease.

Fifth, multimodal integration is essential for accurate diagnosis. No single modality—endoscopy, CT, enterography, or histology—is sufficient alone. The diagnosis of UGI‐CD requires the systematic integration of clinical, endoscopic, radiographic, and histological findings.

Sixth, histologic confirmation is the diagnostic cornerstone. As shown in Figure 6, colonoscopy revealed nodular protuberances at the terminal ileum, and biopsy confirmed non‐caseating granulomas—a pathognomonic feature of CD. This finding underscores that endoscopic findings alone are insufficient; tissue diagnosis is essential, particularly when other modalities are equivocal.

### 3.13 Biologic Therapy Implications

Due to the rarity of UGI‐CD and the absence of standardized treatment protocols, clinical management remains challenging. In both cases, the patients developed obstructive symptoms refractory to conventional first‐line therapies.

#### 3.13.1 Evidence Limitations and Historical Context

Before discussing specific treatment selections, we acknowledge important limitations in the available evidence. To date, no randomized controlled trials have specifically evaluated biologic therapies for isolated UGI‐CD. Most data are extrapolated from studies of luminal CD. Furthermore, no head‐to‐head comparative studies exist among different biologics specifically for duodenal involvement.

Importantly, the clinical decisions described in this report were made in 2020. At that time, the biologic landscape for CD differed from the present. Ustekinumab had been approved for CD in 2016, but real‐world experience for upper GI involvement was very limited. Vedolizumab had limited data for stenotic phenotypes. Upadacitinib was not yet approved for CD. Infliximab, by contrast, had over two decades of established efficacy and safety data.

#### 3.13.2 Rationale for Infliximab in Our Cases (2020 Clinical Context)

We selected infliximab as the initial biologic therapy for reasons relevant to our specific patients and the available evidence in 2020. First, both patients presented with obstructive symptoms requiring rapid symptom relief. A study showed that 58% of patients respond within 2 weeks after a single infusion of infliximab [45]. Second, for CD with stenotic complications, the ECCO guidelines available at the time recommended infliximab for moderate to severe active disease [46]. Third, a randomized controlled trial demonstrated that patients receiving scheduled maintenance therapy achieved significantly higher rates of complete mucosal healing [47].

#### 3.13.3 Alternative Biologic Options (Contemporary Perspective)

While infliximab was a reasonable choice in 2020, we acknowledge that the current biologic landscape offers more options. Ustekinumab (IL‐12/23 inhibitor) has demonstrated efficacy in luminal CD. Vedolizumab (anti‐integrin antibody) is gut‐selective, though data for upper GI involvement remain limited. Adalimumab (subcutaneous anti‐TNF) offers home administration. Upadacitinib (Janus kinase inhibitor) has recently shown efficacy but was not approved for CD at the time of our cases.

In the absence of comparative data, the choice among biologics should be guided by disease phenotype, urgency of symptom control, prior treatment history, and patient preference.

#### 3.13.4 Endoscopic and Surgical Alternatives

Biologic therapy is not the only option for UGI‐CD. Endoscopic balloon dilation is a reasonable option for short (< 5 cm) single strictures without active inflammation. Case series have reported technical success rates of 70%–90% for UGI‐CD strictures, though recurrence is common (30%–50% within 1–2 years). In Case 1, dilation was performed without biopsy or concurrent biologic therapy, which likely contributed to treatment failure. We propose that dilation should be accompanied by deep biopsies and followed by biologic therapy.

Surgical resection is reserved for patients with long, multiple, or complex strictures, those who fail medical therapy, or those presenting with complications. In Case 1, surgery was ultimately required due to the 47‐month diagnostic delay. Earlier diagnosis and biologic therapy might have prevented this outcome, as illustrated by Case 2.

#### 3.13.5 A Balanced Approach to Treatment Selection

Based on the available evidence and the historical context of our cases (2020), we propose the following balanced approach. For patients with an inflammatory phenotype and mild symptoms, conventional therapy may be sufficient. For those with moderate to severe inflammatory disease, biologic therapy is indicated. For patients with a short stricture without active inflammation, endoscopic balloon dilation combined with concurrent biologic therapy is reasonable. For those with long or multiple strictures, surgical referral should be considered.

In our cases treated in 2020, infliximab was a reasonable choice. However, we do not claim that infliximab is superior to other biologics for UGI‐CD, as comparative data are lacking. Clinicians treating similar patients today have more options and should consider multiple factors.

These cases demonstrate that early initiation of potent biologics can be effective for managing complex, treatment‐refractory UGI‐CD. However, treatment decisions must be individualized and contextualized within the available evidence at the time of treatment.

## 4 Recommendations for Early Diagnosis

Based on our literature review and insights from these challenging cases, we propose maintaining a heightened index of suspicion and adopting a structured diagnostic approach for the early detection of UGI‐CD. The following are high‐index clinical scenarios: (1) Unexplained, refractory upper abdominal symptoms (e.g., pain, nausea, vomiting) that persist despite conventional therapy (e.g., proton pump inhibitors). (2) *H. pylori*‐negative duodenal ulcers or those with an atypical morphology (e.g., longitudinal). (3) Young patients presenting with symptoms of gastric outlet obstruction without an identifiable cause. (4) Patients with an established diagnosis of CD elsewhere in the GI tract who develop new UGI symptoms.

Diagnostic steps: (1) Upper endoscopy with mandatory deep biopsies. Perform esophagogastroduodenoscopy with meticulous inspection. Critically, deep biopsies must be obtained during every endoscopic procedure in patients with refractory, *H. pylori*‐negative duodenal ulcers, regardless of whether the primary intent is diagnostic or therapeutic (e.g., dilation). Biopsies should be taken from both abnormal and normal‐appearing mucosa (≥ 6 biopsies). As illustrated in Case 1, failure to obtain biopsies can lead to catastrophic diagnostic delays and preventable surgery. (2) Cross‐sectional enterography (CTE or MRE). This is crucial to evaluate for mural inflammation, stenosis, and fistulas, and to assess the entire small bowel. MRE is preferred to avoid radiation exposure, especially in young patients. (3) Ileocolonoscopy. Perform ileocolonoscopy to rule out concomitant colonic or ileal involvement (Figure 7).

Critical reminder: When endoscopic and imaging findings diverge (e.g., severe stenosis on endoscopy but negative imaging), do not exclude CD. Lower the threshold for repeat endoscopy with biopsies, small‐bowel capsule endoscopy, or magnetic resonance enterography.

## 5 Conclusion

Timely detection of UGI‐CD remains challenging. Based on the two cases presented, this report offers several observations that may inform clinical practice, though they should be interpreted with caution given the descriptive nature of this case series.

First, we observed an exceptionally prolonged 47‐month diagnostic delay in one patient, which appeared to correlate with stricturing complications and subsequent surgical intervention. Second, we identified a notable endoscopic‐radiologic mismatch—severe duodenal stenosis in the absence of typical IBD imaging findings—which suggests that clinicians should not exclude CD based solely on negative initial radiology. Third, we propose a practical diagnostic algorithm to guide the evaluation of refractory, *H. pylori*‐negative duodenal ulcers with stenosis. This algorithm represents a proposed framework based on our experience and literature review; it has not been prospectively validated and should be applied with clinical judgment.

These observations underscore that a high index of suspicion, prompt endoscopy with deep biopsies, and cross‐sectional imaging are critical. When endoscopic and imaging findings diverge, lower thresholds for re‐biopsy or small‐bowel evaluation may be warranted. Early biologic therapy (e.g., infliximab) can induce remission and prevent surgery, as illustrated by Case 2, although this observation derives from a single case and requires confirmation in larger studies.

Future research should focus on standardized diagnostic guidelines incorporating clinical, serologic, and imaging parameters, as well as large‐scale multicenter studies to validate early biologic efficacy and identify predictive biomarkers for disease progression.

While these findings are derived from only two cases and cannot be generalized, they illustrate the potential consequences of diagnostic delay and the possible benefits of early recognition in UGI‐CD. The observations presented here are hypothesis‐generating and intended to raise awareness and stimulate further investigation.

## Author Contributions

Cheng Lan took a major lead in study conception and design, article review, critical revision of the article for important intellectual content, and final manuscript approval. Chaochao Chen and Yongrong Li played a major role in the acquisition and interpretation of the literature and drafting and finalizing the manuscript. Yiyao Chen and Zhoutao He took part in the acquisition and interpretation of the cases. All authors reviewed the manuscript.

## Ethics Statement

This case report was approved by the Medical Ethics Committee of Hainan General Hospital (Approval No.: 2025‐614). Written informed consent was obtained from both patients for publication.

## Conflicts of Interest

The authors declare no conflicts of interest.

## Data Availability

All data generated or analyzed during this study are included in this published article.
